# The Demographic Profile of Suicidal Hanging Deaths in North India

**DOI:** 10.7759/cureus.30409

**Published:** 2022-10-17

**Authors:** Nirmal Nagar, Binaya K Bastia

**Affiliations:** 1 Forensic Medicine and Toxicolgy, All India Institute of Medical Sciences, Rishikesh, Rishikesh, IND; 2 Forensic Medicine and Toxicology, All India Institute of Medical Sciences, Rishikesh, Rishikesh, IND

**Keywords:** suicide, season, death, autopsy, hanging

## Abstract

Background: Suicidal hanging is the most prevalent means of suicide worldwide, particularly among young people, and reveals the state of mental health in certain indigenous populations. According to the National Crime Records Bureau, hanging was the most frequent cause of suicide in India in 2019 and 2020, accounting for 53.6% and 57.8% of total suicidal deaths.

Aim: The current study examines the seasonal distribution of suicide-hanging fatalities in Rishikesh, Uttarakhand, as well as the male and female incidence.

Methods: A four-year retrospective examination of autopsy data at the All India Institute of Medical Sciences (AIIMS), Rishikesh, from October 2018 to July 2022. A total of 1720 autopsies were performed during this period with 130 (7.56%) suicidal hangings.

Results: Males were disproportionately impacted (n=100, 76.92%). The ratio of men to women is 3.33:1. The mean ages of the males and females were 33.09 ± 12.59 and 24.9 ± 7.84 years, respectively. The majority of deaths occur in the third decade of life. The summer months saw the highest number of deaths (April-June).

Conclusion: This data may be used to identify persons with a higher chance of committing suicide by hanging and can be utilized to help people through a nationwide suicidal prevention program that employs a multi-disciplinary team approach. Epidemiological studies should evaluate the psychosocial characteristics of men and women separately to identify the population at risk and develop preventative approaches.

Limitations: This is a single-center, retrospective study.

## Introduction

Suicide is a serious public health concern and a leading cause of death worldwide [1]. The distribution of suicide fatalities reveals information about a community's mental and personal health [2]. In India, 1,53,052 suicides were recorded in 2020, a 10.0% increase over 2019 and an 8.7% increase in the suicide rate from the previous year. In 2020, Uttarakhand had the highest percentage (82.8%) increase in suicides over 2019. Suicide was most commonly committed by hanging (57.8%), poisoning (25.0%), drowning (5.2%), and burning (3.0%) [3]. Scientific studies from Vellore on verbal autopsies emphasize the issue of failing to report suicides in India, highlighting the potential that the situation will worsen [4].

The method of suicide is based on the availability of tools, knowledge of lethal consequences, and the victim's choice. Suicide techniques employed by males and females differ in several ways [5]. In developing countries like India, hanging is the most common method of suicide [6]. The grounds for selecting this method of suicide are (a) the ease with which ligature may be obtained; (b) the high success rate; (c) the various natural and artificial structures from which one might hang oneself; (d) seclusion; (e) painless and quick death; and (vi) bloodless death [7].

Suicidal hanging is defined as a form of asphyxia death produced by the body being self-suspended by a ligature material around the neck, with the body's weight serving as the compression force around the neck. Usually, rope or readily accessible clothing serves as the ligature material. The hanging may cause death by various processes that function separately or in combination. These include spinal cord injury, venous and arterial blockage, airway obstruction, carotid complex compression resulting in reflex cardiac arrest, and so on [8].

According to Kanchan et al., 4.5% of all autopsied cases in the Manipal region of South India involved suicide by hanging; 34.3% of the victims were between the ages of 21 and 30. It was a 2:1 male-to-female ratio. The male and female mean ages were 40.62 and 29.96, respectively. The summer months had the highest mortality rates [9]. In the Delhi and National Capital Region, suicide by hanging constituted 2.67% of all autopsied cases, according to Anand et al. Ages, 21 to 30 made up 42.62% of the victims. The ratio of men to women was 1.7:1 [10].

Hanging is the second most frequent method of suicide deaths. With ages ranging from 12 to 68, there were more females than males. Males committed suicide at an average age of 32.5 +13.6 years, and females at an average age of 25.9 +8.7 years. One-fourth of men were 41 or older, compared to only around 5% of women in that age range. Another considerable discrepancy was observed in the 21-30 age range, with around 60% females to just 35% men [11].

Suicide has several causes, making treatment and prevention challenges. Gender disparities must be considered while creating effective methods. Rishikesh is a rural township in Uttarakhand, Dehradun district in Northern India, and AIIMS Rishikesh is the state's foremost teaching institute. This retrospective study aims to look at the demographic profile of suicide-hanging victims in this region of India. It also examines the gender inequalities between males and females, which may be useful in identifying those at risk and developing preventive methods.

## Materials and methods

Study design and population

A retrospective descriptive study was done utilizing data collected between October 1, 2018, and July 31, 2022. The study comprised all autopsies performed at AIIMS Rishikesh Mortuary between October 1, 2018, and July 31, 2022, where the cause of death was suicidal hanging. A postmortem investigation is required when a death happens abruptly, unexpectedly, suspiciously, or unnaturally. All hanging deaths are classified as unnatural and must undergo an autopsy. A thorough victim profile was developed using autopsy data and details from inquest records provided by the police. Using Microsoft Excel and the Statistical Package for Social Sciences (SPSS) for Windows, version 22.0 (IBM Corp., Armonk, NY), the data for men and women were examined and compared.

## Results

Total (N=1720) autopsies were performed at the AIIMS Rishikesh mortuary during the study period. Suicidal hanging deaths accounted for 7.56% (n=130) of all autopsied cases. Males made up most of the victims (n=100; 76.92%). The ratio of men to women was 3.33:1. Table 1 displays the annual distribution of suicidal-hanging deaths.

**Table 1:** The annual distribution of suicidal hanging deaths.

Year	Total autopsies	Suicidal hangings	Percentage (%)	M/F (ratio)
2018 (Oct-Dec)	49	1	2.04	1:0
2019	435	37	8.50	30/7 (4.28:1)
2020	353	41	11.61	32/9 (3.5:1)
2021	505	34	6.73	25/9 (2.78:1)
2022 (Jan to July)	378	17	4.50	12/5 (2.4:1)
Total	1720	130	7.56	100/30 (3.33:1)

The victim's ages ranged from 12 to 70 years, with a peak incidence in the third decade (n=53, 40.76%) of life, followed by a progressive drop up to the seventh decade. Age groups in the second through fifth decades were the most severely impacted, accounting for 91.54% (n=119) of all suicidal hanging deaths. The victim's mean age (S.D.) was 31.2 ±12.16 years. Males' ages ranged from 16 to 70 in a comparative gender perspective, whereas female ages ranged from 12 to 45. The mean (S.D.) age of men was 33.09 ± 12.59, compared to 24.9 ± 7.84 for women. Men were more exposed in their third and fourth decades, whereas women were more exposed in their second and third decades (Table 2).

**Table 2:** Suicidal hanging deaths by age group

Age groups	Male (n), %	Female (n, %)	Total (n, %)	M/F (ratio)
<19	11, 11%	9, 30%	20, 15.38%	11/9(1.22:1)
20-29	38, 38%	15, 50%	53, 40.76%	38/15(2.53:1)
30-39	23, 23%	4, 13.33%	27, 20.77%	23/4(5.75:1)
40-49	17, 17%	2, 6.67%	19, 14.61%	17/2(8.5:1)
50-59	5, 5%	0	5, 3.84%	5:0
60-69	5, 5%	0	5, 3.84%	5:0
70-79	1, 1%	0	1, 0.76%	1:0
Total	100, 100%	30, 100%	130, 100%	100/30(3.33:1)

**Figure 1:**
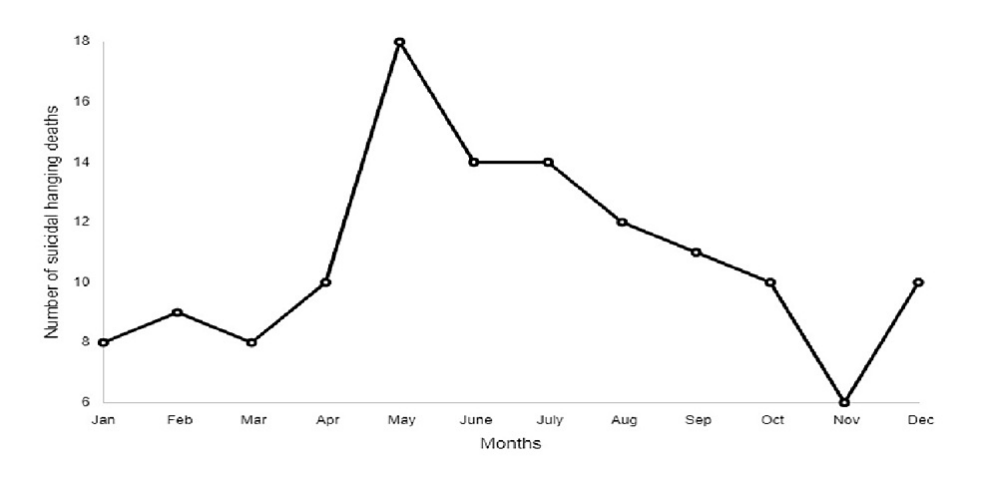
Month-wise distribution of hanging fatalities

Suicidal hanging deaths peaked in the summer (n=42, 32.3%), then fell to their lowest level in the autumn (n = 16, 12.3%). Figure 1 displays the monthly distribution of suicidal-hanging deaths.

**Figure 2:**
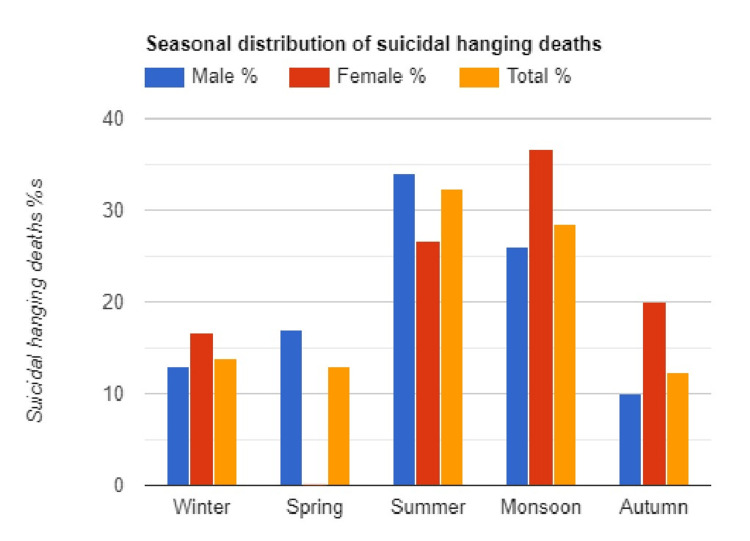
Suicidal hanging fatalities were distributed seasonally.

Suicidal hanging was highest (n=34, 34%) among men during the summer season (April-June) and lowest (n=10, 10%) during the autumn season (October-November). The monsoon season (July to September) had the highest number of female suicidal hanging deaths (n=11, 36.66) and the lowest number (n=0) during the spring season (February-March). Suicidal hanging fatalities were distributed seasonally (Figure 2).

## Discussion

Different racial groups and geographical locations tend to adopt distinct suicide techniques [12]. Values derived from tradition, faith, and civilization appear to play a substantial effect [13]. The growing prevalence of suicide deaths is attributed to several complicated risk factors interplay [14]. Psychiatric disorders, alcoholism, interpersonal turmoil or broken or troubled relationships, issues with the law or the workplace, and financial troubles are a few reasons [15]. The low prevalence of depression in India is ascribed to the population's resistance to seeking treatment at a mental health facility as well as a lack of qualified psychiatrists [16].

Males outnumbered females in our study, which might be explained by the fact that men face greater external challenges, such as higher stress levels and financial obligations. Other researchers' findings concur with our conclusion [5,12,13,17,18]. Only a small number of research indicated that the ratio of female to male cases was equal or marginally in favor of the female [2,11,19].

Similar to other studies conducted worldwide, the largest incidence occurs most commonly during the third and fourth decades and is frequently attributed to the extreme stress a person experiences at this time [18,20]. The youngest victim in our research was 12 years old, while the youngest instance was nine years old [21]. Our study's oldest victim was 70 years old, which is 26 years younger than the oldest case ever documented [22].

Suicidal hanging was more common among young females, accounting for half of the female fatalities in the third decade, followed by the second decade. This age group may have a higher likelihood of suicide due to the increased stress that a woman is likely to feel before and after marriage. The rising financial stress is mostly to blame for the increased prevalence of suicide hanging in males in their third decade, which contributes to 38% of male suicides, followed by the fourth decade of life. The incidence of hanging suicide increases as males age. Males over the age of 70 tend to have more depression than younger males, which can be due to both their rising financial dependency and the medical conditions they are more prone to [9]. Stress levels varied with age and gender. While more women were affected by acute triggering events, more men were affected by chronic stress. While elderly patients experienced more chronic stress, younger participants experienced more acute triggering events before death [23]. In 2020, the maximum number (n=41. 11.61%) of suicide-hanging cases were recorded in our analysis, as suicidal rates increased during the COVID-19 pandemic, as documented in previous studies [24,25].

Suicide seasonal disparity is a long-recognized phenomenon, and there may be a relationship between the seasonal and monthly pattern of successful and failed suicide attempts. According to South African research, maximum hangings were recorded in November and the lowest in September [21]. The number of violent and nonviolent suicide attempts varied by season; for both sexes, violent attempts peaked in spring/autumn, whereas nonviolent attempts peaked in spring/summer [26]. violent suicides have a seasonal pattern, but not nonviolent suicides [27]. Similar to previous research on the Indian population, the summer months witnessed the largest incidence of suicidal hangings [9]. Latitude and environmental factors such as precipitation and sunlight have been observed to influence mood [28,29]. Circadian serotonin cycles in the brain have been linked to the biological basis of seasonal susceptibility [30].

Limitations

This is a single-center retrospective study, and information regarding victims' mental health variables such as known psychiatric disease, previous suicidal thoughts, suicidal attempts, and similar incidents in the family was not considered.

## Conclusions

Based on the multiple criteria examined, the victim profile of suicidal hanging deaths for males and females is as follows: (1) Out of the total, 7.56% of all autopsied cases involved suicide by hanging, (2) Males were affected more than three times as much as females, (3) Males and females aged 20 to 29 were more likely to commit suicide by hanging, (4) Both genders have seasonal asymmetry, with summer months seeing the highest total mortality rates.

Stress management measures and mental health counseling are urged to alleviate the tension produced by outmoded stereotypes to prevent these suicide victims from hanging. The findings support that psychological factors should be included separately in scientific research for men and women to identify risk groups and develop preventive interventions.

Future recommendations: Comprehensive research is required to identify those at high risk of suicide, understand the factors that lead to suicide, and investigate the root of the issue to stop suicidal behavior. Suicidal behavior should be investigated for personal and social reasons, and preventative measures should be implemented. We expect that this study should serve as a database for future research.
